# Bevacizumab in recurrent epithelial ovarian cancer: real-world experience from a tertiary cancer hospital in India

**DOI:** 10.3332/ecancer.2025.1897

**Published:** 2025-04-23

**Authors:** Ranti Ghosh, Debarshi Lahiri, Debjit Ghosh, Kushal Sen, Debanjan Chakraborty, Tapas Maji, Suparna Mazumder, Ranajit Mandal, Arit Bhattacharjee, Jayanta Chakrabarti

**Affiliations:** 1 Department of Radiation Oncology, Chittaranjan National Cancer Institute, 37, SP Mukherjee Road, Kolkoata 700026, India; 2 Department of Radio-Diagnosis, Chittaranjan National Cancer Institute, 37, SP Mukherjee Road, Kolkoata 700026, India; 3 Department of Gynae-Oncology, Chittaranjan National Cancer Institute, 37, SP Mukherjee Road, Kolkoata 700026, India; 4 Department of Surgical Oncology, Chittaranjan National Cancer Institute, 37, SP Mukherjee Road, Kolkoata 700026, India

**Keywords:** bevacizumab, recurrent, epithelial ovarian cancer, outcome

## Abstract

**Background:**

In combination with chemotherapy, bevacizumab, a humanised monoclonal antibody against angiogenesis, significantly increases progression-free survival (PFS) in recurrent epithelial ovarian cancer (EOC). However, due to financial constraints, real-world experience with bevacizumab in EOC is lacking in Indian populations. This study assessed bevacizumab’s efficacy with chemotherapy in platinum-sensitive and resistant EOC in resource-limited Indian populations.

**Method and materials:**

This retrospective study was conducted at a regional cancer hospital in eastern India. Platinum-sensitive and resistant recurrent EOC patients were enrolled between 2021 and 2024. Patients’ demographic and treatment details were retrieved from hospital medical records. All patients received bevacizumab 7.5 mg/kg IV dose with chemotherapy followed by maintenance till disease progression or inadvertent toxicity occurred. Primary endpoints were PFS and objective response rate (ORR); secondary endpoints were overall survival (OS) and safety. Kaplan–Meier plot generated PFS and OS survival curves.

**Results:**

48 patients were enrolled. With a median follow-up of 37 months, 46% of patients progressed on bevacizumab. The median duration of PFS was 17 months (95% CI, 14.31–19.68); it was slightly higher in platinum-sensitive patients at 18 months (95% CI, 14.25–21.74). Half of the patients achieved partial response, with an ORR of 66%. Median OS was not reached due to fewer events. The 3-year OS rate was 83%. About 15 patients who progressed on bevacizumab were able to receive further chemotherapy lines. No new safety concerns were noted. Only 4.2% of patients developed grade 3 proteinuria, one developed arterial thrombosis and two had grade 3 thrombocytopenia. Only one patient died due to a GI fistula.

**Conclusion:**

Bevacizumab plus chemotherapy followed by bevacizumab maintenance till disease progression significantly improved PFS in recurrent EOC. This real-world finding suggests a crucial insight into effective treatment options for financially compromised Indian populations with recurrent EOC.

## Introduction

According to GLOBOCAN 2020, epithelial ovarian cancer (EOC) is the eighth most common cancer and the eighth leading cause of cancer-related death among females globally [1]. EOC is emerging as the third most common malignancy affecting Indian women, after cervical and breast cancer. The annual incidence of EOC is anticipated to rise by 55%, and mortality will increase by 67% by 2035 [2, 3]. It is the most lethal gynaecological malignancy and is associated with a worse prognosis. More than 70% of patients present in the advanced stage with poor long-term outcomes [4]. The standard treatment approach in advanced EOC is cytoreductive surgery followed by systemic therapy with platinum doublet. Despite an initial complete response, most advanced EOC patients suffer from recurrence; among them, 25% of patients become platinum resistant. Treating recurrent EOC is very challenging and associated with the worst prognosis. Re-treatment with various chemotherapy regimens (platinum doublet or single agent), depending upon platinum sensitivity, produced minimal survival benefit; median progression-free survival (PFS) was 7–13 months [5–7]. A newer approach to improve survival is to combine chemotherapy with poly (ADP-ribose) polymerase (PARP) inhibitors plus or minus bevacizumab.

Bevacizumab, a humanised monoclonal antibody, blocks all known isomers of vascular endothelial growth factor A (VEGF-A) and suppresses tumour angiogenesis [8]. Many large-scale randomised control trials (RCTs) evaluated the effectiveness of bevacizumab with chemotherapy in recurrent EOC in platinum-sensitive and resistant settings [9, 10]. The addition of bevacizumab significantly prolongs PFS in those cases. However, these studies primarily included Western patients with very stringent enrolment criteria. Though EOC is a rising trend in India, real-world data using bevacizumab in recurrent EOC has been lacking till now. Due to higher treatment costs, the bevacizumab dose (15 mg/kg body weight) used in those trials is challenging to replicate in India. The ICON7 trial [11] combined 7.5 mg/kg of bevacizumab with chemotherapy in first-line EOC treatment and significantly improved PFS. This study was designed to assess the efficacy and safety of bevacizumab (7.5 mg/kg body weight) in combination with chemotherapy in recurrent EOC in a government hospital’s socioeconomically compromised population.

## Methods and materials

We conducted this retrospective single-arm observational study in the Radiation Oncology department of Chittaranjan National Cancer Institute, Kolkata. The Institutional Ethical Committee (IEC) approved the study protocol (CNCI-IEC-RGI-2024-110) with a consent waiver. We collected retrospective patient data from hospital medical records dated from April 2021 to March 2024. We included all patients over 18 years old with histopathologically confirmed EOC that had clinico-radiologically recurred or progressed after at least one line of platinum doublet-based chemotherapy with good performance status and preserved organ function in the study. Inoperable and up-front metastatic patients were also included. Patients received a chemotherapy regimen (depending upon platinum sensitivity) of up to six cycles in combination with bevacizumab 7.5 mg/kg intravenous 3 weekly until disease progression or inadvertent toxicities occurred. The platinum-free interval (PFI) was defined as the time from the last platinum treatment to the detection of recurrence [12]. A PFI of more than 6 months was considered to be a platinum-sensitive disease. A PFI of less than 6 months was considered platinum-resistant and patients who progressed with platinum therapy were considered to have platinum refractory disease. Three platinum-based regimens (Paclitaxel-Carboplatin, Gemcitabine–Carboplatin and Pegylated Liposomal Doxorubicin-Carboplatin) were used in platinum-sensitive disease. In contrast, single-agent chemotherapies such as Pegylated liposomal doxorubicin, Gemcitabine and Capecitabine were used in platinum-resistant disease in combination with bevacizumab. Patients with inadequate medical records, those who defaulted during bevacizumab or received less than 3 months of bevacizumab were excluded from the study. Patients with a history of bowel obstruction due to ovarian malignancy or other diseases, previous history of GI fistula, perforation, intraabdominal abscess, clinico-radiologically rectosigmoid invasion, uncontrolled hypertension, major abdominal surgery within 4 weeks, history or active thrombotic or haemorrhagic disease within 6 months of the study’s initiation, active cardiovascular diseases or a persistent nonhealing wound were excluded from the study. Patients received bevacizumab from either a state insurance scheme or a hospital patient fund. Patients were evaluated every 3 months using contrast-enhanced computed tomography of thorax, Abdomen and Pelvis and response was recorded by the Response Evaluation Criteria in Solid Tumours v1.1. Toxicities related to bevacizumab therapy (i.e., hypertension, proteinuria, GI fistula, perforation, thromboembolic events, thrombocytopenia and conservatively managed subacute intestinal obstruction (SAIO)) were recorded and graded according to the Common Terminology Criteria for Adverse Events v5.1.

Patients’ demographic details, medical and treatment history, response, progression or death data were collected from hospital medical records and telephonic conversations with patients or relatives and followed up till September 2024. We aimed to assess bevacizumab’s effectiveness in recurrent ovarian cancer patients along with standard chemotherapy regimens in a real-world scenario. The primary objectives were objective response rate (ORR) and PFS; overall survival (OS) and bevacizumab’s safety profile were secondary endpoints. ORR is defined as the percentage of complete and partial response to bevacizumab within a specified study period. PFS was defined as the interval from initiation of the bevacizumab with chemotherapy in recurrent EOC till progression or death due to any cause. OS was calculated as the time period between the date of diagnosis to death from any cause.

Demographic, clinical and treatment variables were analysed by using descriptive statistics. PFS and OS were estimated by the Kaplan–Meier plot and Cox regression method. Lost to follow-up patients were contacted telephonically and their outcomes were included in OS and PFS analysis. Statistical analysis was done on Stata Statistical Software: Release 13 (2013; Stata Corp LLC, College Station, TX).

## Results

48 patients were recruited in this study. The median age of this cohort was 47.5 years. Around half of the patients were in the 41–50 age group; only two (4.2%) patients were above 60. Most patients (62.5%) had performance status ECOG 1 at the treatment’s initiation; only 10% were ECOG 2. The most common histopathology was high-grade serous carcinoma (73%); only one mucinous carcinoma patient was included. Stages III and IV patients were included at similar frequencies. Platinum-sensitive patients accounted for 77.1%; six patients (12.5%) were platinum-resistant and five patients (10.4%) were platinum-refractory. Around 79% of patients underwent primary or interval cytoreductive surgery. Patients who had received previous single-line chemotherapy accounted for 83%. The rest of the patients had received the previous two lines of chemotherapy. The most common platinum doublet chemotherapy regimen with bevacizumab was pegylated liposomal doxorubicin and carboplatin (37.5%); gemcitabine-carboplatin was used in 22.9% and paclitaxel-carboplatin was used in 16.7%. Single-agent chemotherapy, including gemcitabine, capecitabine and pegylated liposomal doxorubicin, was used in 23% of patients. Table 1 showed baseline characteristic details. The median PFI was 8.5 months. The median duration of the bevacizumab treatment was around nine months (inter quartile range 3–35). Most patients received around 12 cycles of bevacizumab. Around 21% of patients were experiencing co-morbid conditions at bevacizumab’s initiation; among them, 12.5% were hypertensive and 8.3% were diabetic, controlled with medications. Around one-third of patients’ bevacizumab cycles were interrupted because of toxicities and supportive care. Bevacizumab dose reduction was not done.

**Figure 1.**
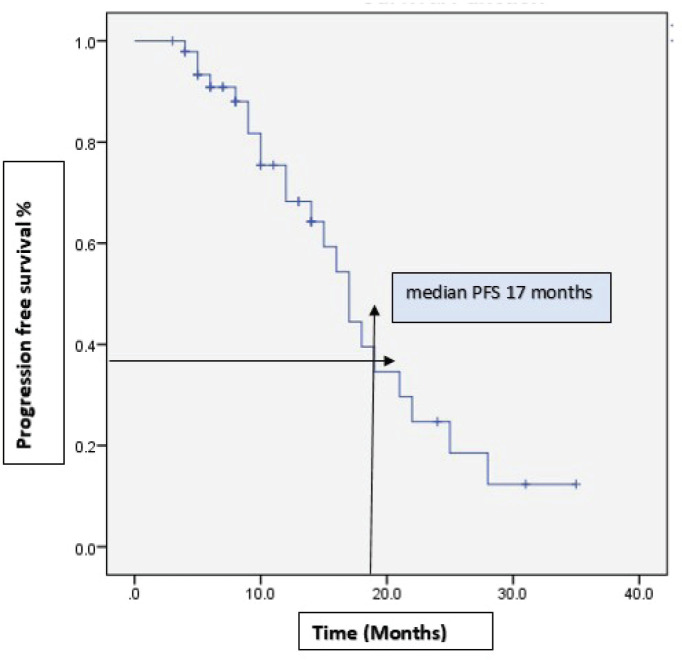
Kaplan–Meir plots show median PFS of 17 months with bevacizumab of the entire cohort.

**Figure 2.**
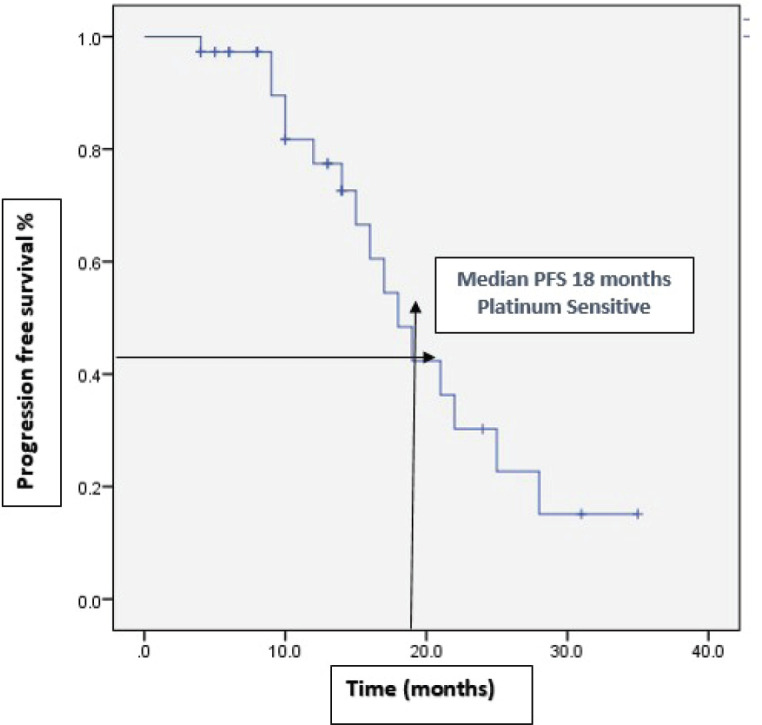
Kaplan–Meir plots demonstrate median PFS of 18 months among platinum sensitive group.

**Figure 3.**
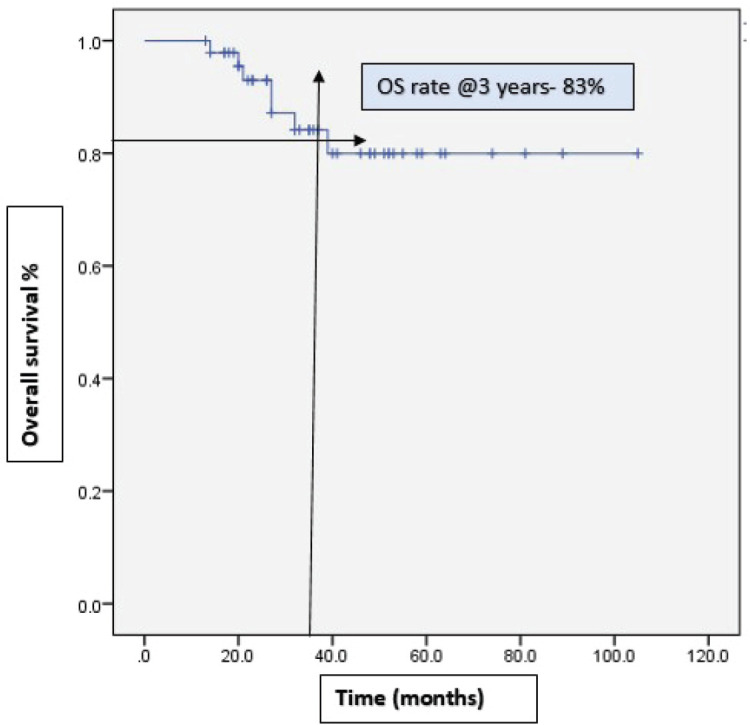
Kaplan–Meir plots of OS rate at 3 years was 83%.

With a median follow-up of 37 months, 46% (22) of the patients progressed on bevacizumab. Among them, 15 patients received further lines of chemotherapy and 7 patients died. The remaining patients (54%) continued bevacizumab. Half of the patients achieved partial response. Complete response was 14.6%, stable disease was 29.2%, with ORR at 66% (Table 2). The median PFS with bevacizumab was 17 months (95% CI, 14.31–19.68), with a higher PFS of 18 months (95% CI, 14.25–21.74) in the platinum-sensitive group. Kaplan–Meir plots for PFS was demonstrated in Figures 1 and 2. Median OS was not reached due to less events. The 3-year OS rate was 83% (Figure 3).

The most common adverse events were proteinuria (70%), hypertension (48%), thrombocytopenia (20%) and SAIO (23%). Most of them were grade 1 toxicities and reversible. Two patients developed beyond grade 2 proteinuria, two developed grade 3 thrombocytopenia, two (4.2%) patients developed a GI fistula and one devolved into an arterial thrombus. No patients suffered from GI perforation or acute obstruction. Toxicities according to grade were summarised in Table 3.

## Discussion

To the best of our knowledge, this study is the first real-world research in India evaluating bevacizumab’s (7.5 mg/kg body weight) effectiveness in combination with chemotherapy to treat recurrent EOC. The primary endpoint of this study was met; the addition of bevacizumab significantly increased ORR and prolonged PFS. ORR was 66%, and the median PFS with bevacizumab was 17 months (95% CI, 14.31–19.68).

High VEGF expression and angiogenesis are the most important promoters of ovarian cancer progression and relapse; both correlate negatively with OS and PFS. Phase II studies documenting bevacizumab’s antiangiogenic activity in recurrent EOC showed improved ORR with good median duration response [13]. Large-scale RCTs in recurrent epithelial ovarian, tubal and peritoneal carcinoma failed to show any significant OS benefit with the addition of bevacizumab along with chemotherapy. The OCEAN trial [9] showed that combining bevacizumab with gemcitabine-carboplatin in platinum-sensitive recurrent EOC improved the median PFS by 4 months compared with the chemotherapy only arm (12.4 versus 8.4 months, with an HR of 0.484 [95% CI, 0.388–0.605; log rank *p* < 0.0001]). However, the GOG-213 trial [14] combined bevacizumab with paclitaxel-carboplatin in platinum-sensitive recurrent EOC but failed to show statistically significant OS improvement. Post hoc analysis identified meaningful increments of median PFS and ORR. Pfisterer *et al* [15] demonstrated that in platinum-eligible recurrent EOC, a pegylated liposomal doxorubicin-carboplatin-bevacizumab regimen resulted in a two-month higher PFS (13.3 versus 11.6 months) than gemcitabine-carboplatin-bevacizumab. The U.S. Food and Drug Administration approved bevacizumab as an antiangiogenic agent for use in platinum-sensitive and resistant recurrent EOC [16].

All large single-centre or multicentre trials mainly included Western patients, a very homogenous population that met strict inclusion and exclusion criteria. The GOG-213 and OCEAN trials included only 14% and 3.7% Asian patients, respectively, in the bevacizumab arm. In developing countries such as India, using bevacizumab in real-world scenarios is limited due to its high treatment cost and poor personal and government insurance coverage. Most patients present with extensive disease recurrence and poor general condition, unable to fit into bevacizumab treatment criteria. Real-world studies are crucial in providing evidence of treatment effectiveness in routine clinical practice. Though generic bevacizumab has been available in India since 2016, the cost of bevacizumab at a dose of 15 mg/kg body weight is more than USD 4,000 for 12 cycles, a significant challenge for most Indian patients. We used a 7.5 mg/kg body weight dose in our patients to cover the cost under state insurance and hospital funds for needy patients.

Our study’s demographic profile was similar to most RCTs and included mainly serous histology (73%) and ECOG 1 (62.5%) patients. Most patients had received a prior one line of chemotherapy and were stages III and IV at presentation. Half of the platinum-sensitive patients received a pegylated liposomal doxorubicin-carboplatin-bevacizumab regimen. The median number of bevacizumab cycles [12] was similar to the OCEAN trial. Our patients’ median PFS was 4–5 months higher than major RCTs, 17 months (95% CI, 14.31–19.68) in the total cohort and 18 months in platinum-eligible patients. Only 11 platinum-resistant patients were included, but their median PFS was 6.5 months, similar to the Aurelia trial [10]. This difference in PFS may be attributed to the difference in recurrent EOC’s prognostic pattern between Asian and Caucasian populations. As per the NRG Oncology/GOG Ancillary study [17], 5 years of disease-specific survival is around 8% higher in the Asian population compared to Caucasians (54.1% versus 46.1%, *p* value 0.001). Real-world experiences with bevacizumab in recurrent EOC in the Asian population are very limited. In a retrospective study, Hung *et al* [18] showed that adding bevacizumab with chemotherapy improved the median PFS compared to chemotherapy alone (18.9 and 9.6 months = 0.070). Similar to our study, this trial used a 7.5 mg/kg body weight dose for more than half of the patients. Another retrospective Chinese study showed lower PFS in platinum-sensitive and resistant patients when treated with multiple lines of chemotherapy before bevacizumab, 11 and 5 months, respectively, than major RCTs [19].

The toxicity profile of this study was similar to other large prospective studies, with hypertension and proteinuria being the most common side effects. However, no patients developed grade 3 hypertension, and the proteinuria incidence of grade 3 or beyond was lower (4.2%), which may be due to the lower bevacizumab dose. One patient developed arterial thrombosis, and two patients developed grade 3 thrombocytopenia with mucosal haemorrhage, similar to Patil *et al* [20] who explored bevacizumab toxicities in the Indian population. Incidences of grade 1 SAIO were higher due to the presence of extensive omental disease, which was managed conservatively. However, toxicities of grade 3 or beyond were lower than historical data; about one-third of patients needed to interrupt treatment for supportive care, financial reasons or social problems. One patient developed a recto-vaginal fistula, which was managed by a diverting colostomy and healed spontaneously. Only one patient died because of a GI fistula causing peritonitis.

Our study’s major limitations were retrospective and concerned the small sample size and short follow-up. The median OS was not reached due to too few events. The study included a heterogeneous patient profile with both platinum-sensitive and resistant diseases. Due to the small sample size, PFS between platinum sensitive versus resistant group was not significant. Due to the cost barrier, neither BRCA gene mutation and/or homologous recombination deficiency testing nor PARP inhibitors were offered to patients, which may have hampered the outcome. The role of secondary cytoreductive surgery was not evaluated in this study. Large, prospective randomised trials should be planned for the most appropriate outcome.

In our study, ORR was 66%, similar to the ICON7 trial, with 15% of patients achieving complete response. More than half of the patients were continuing bevacizumab at the time of data cut-off, and their response was maintained for over 1 year. Two-thirds of patients were able to resume further lines of chemotherapy beyond progression. Despite its limitations, our study provided a meaningful addition to understanding bevacizumab’s role in recurrent EOC in limited-resource, poor socioeconomic populations. Bevacizumab (at the dose of 7.5 mg/kg body weight) combined with chemotherapy can be incorporated into routine clinical practice to minimise cost. It demonstrated a good safety profile with improved clinical outcomes.

## Conclusion

Angiogenesis plays the most important role in the progression and recurrence of ovarian cancer. The antiangiogenic role of bevacizumab is well-established in recurrent EOC. Our study demonstrated that the addition of bevacizumab with commonly used chemotherapy significantly improved PFS and ORR among Indian women with recurrent EOC. It correlated clinical trial data in a real-world scenario. The use of bevacizumab at a dose of 7.5 mg/kg combined with chemotherapy can be a standard approach to treating recurrent EOC in socioeconomically compromised populations and developing countries.

## Conflicts of interest

None declared.

## Funding

No funding for this study.

## Figures and Tables

**Table 1.** Baseline characteristics.

Parameters	*N* = 48 (Percent)
Age	
Median	47 years (Range 31–66)
*ECOG status	
0	13 (27.1%)
1	30 (62.5%)
2	5 (10.4%)
'FIGO stage	
I	1 (2.1%)
III	23 (47.9%)
IV	24 (50%)
Histology	
High grade serous	35 (72.9%)
Mucinous	1 (2.1%)
Others/Adenocarcinoma NOS +	12 (25.0%)
Comorbidities	
Hypertension	6 (12.5%)
Diabetics	4 (8.3%)
Others	5 (10.4%)
Surgery	
No	10 (20.8%)
Yes	38 (79.2%)
Previous chemotherapy line	
First line	40 (83.3%)
Second line	8 (16.7%)
Platinum sensitivity	
Sensitive	37 (77.1%)
Resistant	6 (12.5%)
Refractory	5 (10.4%)
Carboplatin plus bevacizumab	
Peg liposomal doxorubicin	18 (37.5%)
Gemcitabine	11 (22.9%)
Paclitaxel	8 (16.7%)
Others	11 (22.9%)

* ECOG- Eastern Cooperative Oncology Group , ' FIGO- International Federation of Genecology and Obstetrics, + NOS- not otherwise specified

**Table 2.** Response.

Best response (RECIST V1.1)	*N* (Percent)
Complete response	7(14.6%)
Partial response	25(52.1%)
Stable diseases	14(29.2%)

**Table 3.** Toxicity details (CTCAE V5.1).

Parameters	*N* (Percent)
Hypertension	
Grade 1	21(43.8)
Grade 2	2(4.2%)
Proteinuria	
Grade 1	29(60.4%)
Grade 2	3(6.3%)
Grade 3	1(2.1%)
Grade 4	1(2.1%)
Subacute intestinal obstruction	
Grade 1	11(22.9%)
GI-fistula	
Yes	2(4.2%)
Thrombosis (Venous)	
Yes	1(2.1%)
Thrombocytopenia	
Grade 1	6(12.5%)
Grade 2	2(4.2%)
Grade 3	2(4.2%)
